# Influence of atrial ultrastructural remodeling on its early mechanical transport following surgery for atrial fibrillation and mitral insufficiency

**DOI:** 10.1186/1749-8090-8-S1-O63

**Published:** 2013-09-11

**Authors:** T Kopjar, H Gasparovic, M Cikes, V Velagic, Z Colak, LJ Hlupic, M Petricevic, L Svetina, T Fabijanić, D Milicic, B Biocina

**Affiliations:** 1 Department of Cardiac Surgery, University Hospital Centre Zagreb, Zagreb, Croatia; 2 Department of Cardiology, University Hospital Centre Zagreb, Zagreb, Croatia; 3 Department of Anesthesiology, University Hospital Centre Zagreb, Zagreb, Croatia; 4 Department of Pathology, University Hospital Centre Zagreb, Zagreb, Croatia

## Background

Establishment of sinus rhythm (SR) following radiofrequency ablation (RFA) for longstanding persistent atrial fibrillation (AF) is not necessarily an equivalent to physiological atrial mechanical activity. We aimed to determine the influence of atrial ultrastructural remodeling on the recovery of its mechanical transport following restitution of SR.

## Methods

We enrolled 32 patients operated for severe mitral regurgitation. Half of the patients had no history of AF constituting the SR group. The remaining half had concomitant RFA for longstanding persistent AF, the RFA group. Intraoperative transesophageal echocardiography was used for tissue Doppler indices (TDI) data acquisition at the left atrial (LA) lateral wall. During post-procedural data acquisition all patients were in SR. LA biopsies were obtained during surgery and quantified for fibrosis after Mallory’s trichrome staining.

## Results

Atrial mechanical contraction was noted in both groups following surgery, although TDI values of late diastolic strain rate (SRI A’) and tissue velocity (TVI A’) were superior in the SR group (-1.49±1.04 vs. -2.82±1.97, p=0.022; 1.22±1.08 vs. 3.86±2.30, p=0.003). Post-procedural atrial filling was compromised in the RFA (TVI S’ -4.20±2.27 vs. -2.70±1.13, p=0.031) and the SR group (SRI S’ 4.31±3.03 vs. 3.93±2.22, p=0.009). Negative correlation between fibrosis and the pre-procedural SRI A’ was noted in the SR group (r=-0.69, p=0.015), although not significant throughout both groups (r=-0.33, p=0.088).

## Conclusions

We have shown that the restoration of SR following RFA and mitral valve surgery is not equal to normal atrial mechanical transport. Interstitial fibrosis, a symbol of ultrastructural remodeling, is a negative precursor of LA mechanical activity. Our data did not corroborate clear difference in the extent of fibrosis between groups, although a tendency towards more severe ultrastructural remodeling was noticed in patients with history of AF.

